# Digital speech biomarkers can measure acute effects of levodopa in Parkinson’s disease

**DOI:** 10.1038/s41531-025-01045-5

**Published:** 2025-07-01

**Authors:** M. Sousa, P. Krýže, D. Amstutz, K. Petermann, A. Averna, M. Castelli, A. D. Magalhães, A. Jorge, J. Švihlík, T. Tykalová, V. Illner, M. E. Maradan-Gachet, A. A. Diamantaras, J. Waskönig, M. L. Lachenmayer, I. Debove, G. Tinkhauser, T. Nef, J. Rusz, P. Krack

**Affiliations:** 1 Department of Neurology, Bern University Hospital and University of Bern, Bern, Switzerland; 2 Graduate School of Health Sciences, University of Bern, Bern, Switzerland; 3 Department of Circuit Theory, Faculty of Electrical Engineering, Czech Technical University in Prague, Prague, Czech Republic; 4 Department of Biomedical Research, University of Bern, Bern, Switzerland; 5 ARTORG Center for Biomedical Engineering Research, Gerontechnology and Rehabilitation, University of Bern, Bern, Switzerland; 6 Department of Mathematics, Informatics and Cybernetics, Faculty of Chemical Engineering, University of Chemistry and Technology, Prague, Czech Republic

**Keywords:** Motor control, Parkinson's disease

## Abstract

Speech abnormalities in Parkinson’s disease (PD) are heterogeneous and often considered resistant to levodopa. However, human hearing may miss subtle treatment-related speech changes. Digital speech biomarkers offer a sensitive alternative to measure such changes objectively. Speech was recorded in 51 PD patients during ON and OFF medication states and compared to 43 healthy controls matched for language and gender. Acute levodopa effects were significant in prosodic (F0 standard deviation, *p* = 0.03, effect size = 0.47), respiratory (intensity slope, *p* = 0.02, effect size = 0.49), and spectral domains (LTAS mean, *p* = 0.01, effect size = 0.35). Stepwise backward regression identified 8 biomarkers reflecting hypokinetic symptoms, 6 for dyskinetic symptoms, and 7 for medication-state transitions. Hypokinetic compound score correlated strongly with MDS-UPDRS-III changes (*r* = 0.70; MAE = 6.06/92), and the dyskinetic compound score with dyskinesia ratings (*r* = 0.50; MAE = 1.81/12). Medication-state transitions were detected with AUC = 0.86. This study highlights the potential of digital speech biomarkers to objectively measure levodopa-induced changes in PD symptoms and medication states.

## Introduction

Speech is one of the most complex human skills, as it requires not only a finely timed coordination of phonatory, articulatory and respiratory muscles^1,2^, but also a complex interaction of different motor, cognitive and emotional systems^3,4^. As a result, speech is highly susceptible to neuronal damage^5,6^, which explains why more than 90% of Parkinson’s disease (PD) patients develop speech abnormalities at some stage of the disease, globally known as hypokinetic dysarthria^7^. This is characterized by prosody abnormalities (i.e. monopitch and monoloudness), imprecise consonant articulation, speech rhythm abnormalities, dysphonia (i.e. harsh and breathy voice), and ventilatory insufficiency^6–8^. These speech abnormalities are primarily thought to reflect bradykinesia, the hallmark feature of PD^7,9–11^.

While bradykinesia, appendicular rigidity, and tremor improve upon treatment with dopaminergic replacement therapies (DRT)^12,13^, the treatment’s effect on speech has been inconclusive, leading many authors to conclude that speech may be a levodopa-resistant axial symptom^14–22^. However, a recent study has demonstrated a positive long-term effect of DRT in de novo PD after treatment initiation, particularly in improving dysphonia^23^. Nonetheless, demonstration of a robust acute effect has been particularly challenging^19,21,24^.

It is possible that previous studies failed to demonstrate a clear acute effect of the DRT due to lack of sensitivity of the perceptual speech methods used to detect individual speech changes. Perceptual speech assessment is intrinsically subjective, highly dependent on examiner’s experience, and consequently requires multiple highly trained examiners to increase its reliability^25^. Moreover, it has been shown that perceptual assessment is not sensitive enough to distinguish healthy controls speech from patients with prodromal PD and patients with early PD, whereas digital speech analysis allowed such classification with good accuracy^2^.

Another important factor to consider is that during the course of the disease, patients may start to experience long-term side effects from the DRT such as dyskinesia^26,27^, which can worsen some aspects of speech in a seemingly paradoxical way as at the same time akinesia is improved^28^.

In recent years, digital speech analysis has advanced rapidly, and currently it is possible to identify and automatically compute digital speech features that can measure different perceptual speech domains affected in PD with greater sensitivity and granularity^1,29^.

Although wearable devices have enabled objective monitoring of the dopaminergic response of some motor symptoms of PD, such as bradykinesia, tremor, gait or dyskinesia^30–33^, their utility has been mostly confined to predicting tremor and dyskinesia^31,32^. Conversely, their accuracy in measuring bradykinesia response and other axial symptoms such as changes in speech, remains unknown^30–33^. Notably, bradykinesia and axial symptoms, including speech, along with dyskinesia, are the motor symptoms most strongly correlated with quality of life in PD^34^. Thus, there is a clear need for other objective, non-intrusive tools capable of accurately capturing these symptoms.

Therefore, our primary objective was to investigate whether digital speech biomarkers can ascertain subtle speech changes associated with dopaminergic medication. Secondly, we sought to identify the digital speech biomarkers that most accurately reflect changes in the hypokinetic and hyperkinetic motor features of PD across two medication states (ON vs OFF medication). Finally, we evaluated the predictive capacity of the selected motor speech biomarkers to identify medication condition transitions.

## Results

### Baseline characteristics

In this study, we assessed 51 patients with PD exhibiting moderate to severe motor fluctuations and 43 healthy controls matched for language and gender. The PD patients were significantly younger than the healthy controls (*p* = 0.04). No other statistically significant demographic differences were observed between the groups. Detailed demographics and clinical characteristics are presented in Table 1. The demographics and clinical characteristics of a subgroup of 10 patients, who underwent two OFF-medication recordings and one ON-medication recording, are detailed in Supplementary Table 2.

**Table 1:** Baseline and clinical characteristics of PD and healthy controls included

	PD (*n* = 51)	HC (*n* = 43)	*p*-value
**Age (years), mean (sd)**^a^	63.27 (7.94)	66.74 (8.47)	0.04*
**Gender m/f (%)**^b^	41/10 (80.4/19.6)	34/9 (79.1/20.9)	1.0
**Language (%)**^c^			
−German	34 (66.7)	27 (62.8)	
−French	11 (21.6)	10 (23.3)	0.95
−Italian	5 (9.8)	5 (11.6)	
−English	1 (2.0)	1 (2.3)	
**MoCA, mean (sd)**^a^	25.94 (3.67)	26.72 (2.33)	0.62
**Disease duration (years) mean (sd)**	10.98 (3.96)	n.a.	-
**LEDD (mg/d), mean (sd)**	1257.35 (527.66)	n.a.	-
**Levodopa dose given (mg), mean (sd)**	279.80 (50.42)	n.a.	-
**MDS-UPDRS I, mean (sd)**	12.64 (5.21)	n.a.	-
**MDS-UPDRS II, mean (sd)**	15.56 (6.37)	n.a.	-
**MDS-UPDRS III OFF, mean (sd)**	46.06 (12.88)	n.a.	-
**MDS-UPDRS III ON, mean (sd)**	22.31 (11.81)	n.a.	-
**MDS-UPDRS IV, mean (sd)**	9.68 (4.20)	n.a.	-
**Marconi dyskinesia scale OFF, mean (sd)**	0.10 (0.70)	n.a.	-
**Marconi dyskinesia scale ON, mean (sd)**	8.20 (5.11)	n.a.	-

*PD* Parkinson’s disease, *HC* Healthy Controls, *sd* Standard deviation, *MoCA* Montreal Cognitive Assessment, *LEDD* Levodopa equivalent daily dose, *MDS-UPDRS-III* Movement Disorder Society-Unified Parkinson’s Disease Rating Scale, *n.a.* not-applicable, *m* male, *f* female.

^a^independent t-test.

^b^Chi-square test.

^c^Fisher’s exact test.

^*^p < 0.05.

### Speech levodopa responsiveness

**Fig. 1:**
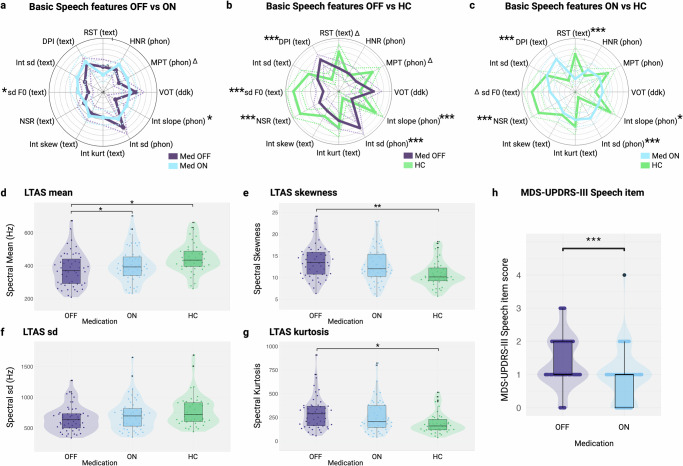
Comparison of individual basic digital acoustic speech biomarkers of Parkinson’s disease (PD) patients in OFF medication in purple, ON medication in blue and Healthy controls (HC) in green. **a** Comparison of PD patients in OFF vs ON medication condition, after z-score normalization. Monopitch (sd F0) and slope of intensity (Int slope) showed a statistically significant difference between both conditions. Maximal phonation time (MPT) demonstrated a nonsignificant trend. **b** PD patients in OFF medication vs HC (adjusted by age, gender and MoCA), after z-score normalization. Monopitch (sd F0), Net speech rate (NSR), duration of pause intervals (DPI), standard deviation of intensity (Int sd) and slope of intensity (Int slope) were significantly different between both groups. **c** PD patients in ON medication vs HC (adjusted by age, gender and MoCA), after z-score normalization. **d–g** different moments of LTAS of PD patients in OFF medication, ON medication and HC. LTAS mean was statistically different between PD patients in ON vs OFF medication conditions. LTAS mean, LTAS skewness and LTAS kurtosis was significantly different in PD patients in OFF state vs HC. **h** MDS-UPDRS-III speech item of PD patients in medication OFF vs medication ON, showing a statistically significant improvement of speech item across medication states. *p < 0.05, **p < 0.01, ***p < 0.001, ^∆^ - non-significant trend. HNR Harmonics-to-noise ratio, VOT Voice to onset time, RST Rate of speech timing, Int kurt Kurtosis of Intensity, Int skew Skewness of intensity, LTAS Long-term averaged spectrum, sd Standard deviation, kurt Kurtosis, skew skewness. The 95% confidence intervales are displayed with dashed lines. Created in https://BioRender.com.

The first objective of this study was to explore the modulation of a set of selected basic speech features representing different speech domains (Fig. 1A-C) and different LTAS moments (Fig. 1D-G) by levodopa in PD. A group of matched healthy controls is also shown to serve as reference values.

After administration of a suprathreshold dose of levodopa, statistically significant improvements in slope of intensity during phonation (Int slope [phon]), monopitch (sdF0), and LTAS mean in the ‘ON’ medication condition was observed. Remarkably, the values of these three variables in the ‘ON’ condition approached those observed in healthy controls (Fig. 1C and D-G), suggesting that these changes represent speech improvement. While the values for monopitch (sdF0) and the slope of intensity (Int slope) significantly differed from those of healthy controls in both medication conditions (Fig. 1B, C), the LTAS mean was significantly different only in the ‘OFF’ condition (Fig. 1D). Importantly, the speech improvement observed from the OFF to the ON medication condition in Parkinson’s disease was not only captured by the digital speech biomarkers but was also perceptible through the MDS-UPDRS-III speech item (Fig. 1H). Additionally, we verified that when patients were assessed 2 times in OFF medication condition (Supplementary Fig. 2), the 2 assessments presented a strong to very strong correlation, supporting that the effects observed in this study between medication OFF and ON are not solely due to repetition effect, lack of stability of speech analysis or order effect.

### Digital speech biomarkers to index hypokinetic symptoms

**Fig. 2:**
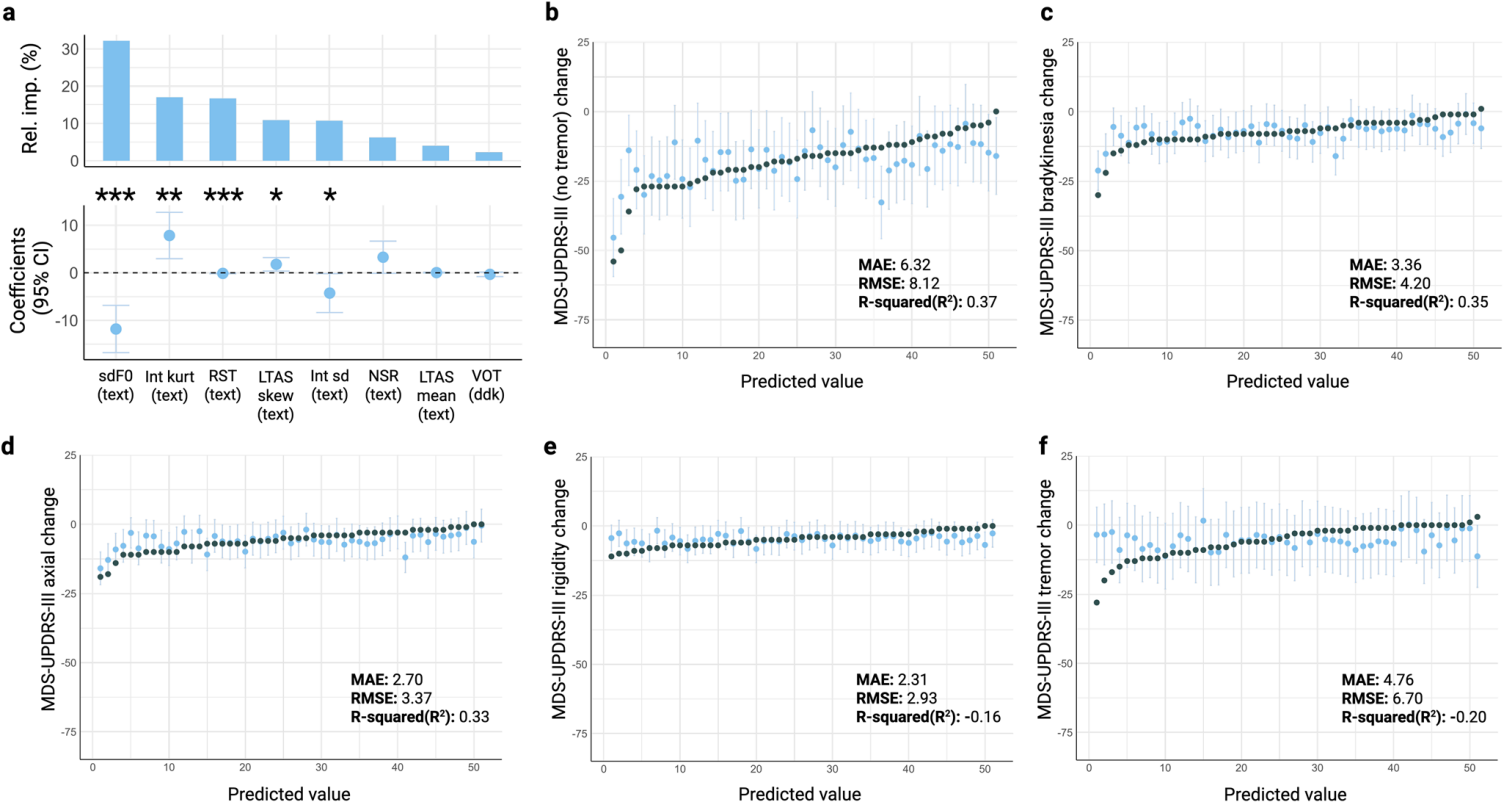
Hypokinetic model for digital speech features selection and accuracy to detect changes in hypokinetic symptoms of PD. **a** Backward stepwise linear regression model plot depicting the model’s relative importances, regression coefficients, and 95% Confidence Intervals to predict changes in hypokinetic symptoms of PD (MDS-UPDRS-III without tremor items). **b** Actual value of the change in MDS-UPDRS-III (without tremor items) [grey] vs the predicted value and respective 95% CI (blue). **c** Actual value of the change in MDS-UPDRS-III bradykinesia subscore (grey) vs the predicted value and respective 95% CI (blue). **d** Actual value of the change in MDS-UPDRS-III axial subscore (grey) vs the predicted value and respective 95% CI (blue). **e** Actual value of the change in MDS-UPDRS-III rigidity subscore (grey) vs the predicted value and respective 95% CI (blue). **f** Actual value of the change in MDS-UPDRS-III tremor subscore (grey) vs the predicted value and respective 95% CI (blue). *p < 0.05, **p < 0.01, ***p < 0.001. VOT Voice to onset time, NSR Net speech rate, RST Rate of speech timing, Int sd Standard deviation of Intensity, Int kurt Kurtosis of intensity, sdF0 standard deviation of fundamental frequency, LTAS Long-term averaged spectrum, sd Standard deviation, kurt Kurtosis, skew Skewness, CI Confidence Interval, MAE Mean absolute error, RMSE Root mean squared error. Created in https://BioRender.com.

In our second phase of analysis, we investigated which digital speech biomarkers most accurately reflect changes in hypokinetic symptoms of PD after levodopa administration. As outlined in Table 2. Figure 2A, the digital speech biomarkers that most effectively explained changes in hypokinetic symptoms of PD, ranked by descending relative importance, included: standard deviation of fundamental frequency (monopitch, sd F0 [text]), intensity kurtosis (Int kurt [text]), rate of speech timing (RST [text]), LTAS skewness (text), standard deviation of intensity (monoloudness, Int sd [text]), net speech rate (NSR [text]), LTAS mean (text), and voice to onset time (VOT [ddk]). Collectively, these variables accounted for 60% of the observed variability.

**Table 2:** Stepwise backward selection regression model to predict changes in hypokinetic and hyperkinetic symptoms of PD, ordered by relative importance

	Estimate	*p* value
**HYPOKINETIC FEATURES MODEL** (dependent variable: MDS-UPDRS-III (no tremor) change)		
INTERCEPT	− 16.84165	<0.001 ***
sdF0 (text) change	−11.80804	<0.001 ***
Int kurt (text) change	7.84045	<0.01 **
RST (text) change	−0.11860	<0.001 ***
LTAS skew (text) change	1.78596	<0.05 *
Int sd (text) change	−4.26142	<0.05 *
NSR (text) change	3.27815	0.0573
LTAS mean (text) change	0.04163	0.1914
VOT (ddk) change	−0.32521	0.1679
*Residual std. error: 7.118 (42 df); Multiple R2: 0.60; Adj. R2: 0.52; F-statistic: 7.9; p-value:* < *0.001*		
**HYPERKINETIC FEATURES MODEL** (dependent variable: Marconi (axial subscore) change)		
INTERCEPT	2.479927	<0.001 ***
MPT (text) change	0.189965	<0.05 *
Int sd (text) change	0.685253	0.0662
Int sd (phon) change	-0.627082	0.0702
LTAS sd (text) change	0.007723	<0.05 *
LTAS skew (text) change	0.992226	<0.05 *
LTAS kurt (text) change	-0.009829	0.1262
*Residual std. error: 2.078 (44 df); Multiple R2: 0.37; Adj. R2: 0.29; F-statistic: 4.3; p-value:* < *0.01*		

*VOT* Voice to onset time, *NSR* Net speech rate, *RST* Rate of speech timing, *Int sd* standard deviation of intensity, *Int kurt* Kurtosis of intensity, *sdF0* Standard deviation of fundamental frequency, *MPT* Maximal phonation time, *LTAS* Long-term averaged spectrum, *sd* Standard deviation, *kurt* Kurtosis, *skew* Skewness.

*p*-value significance—*p < 0.05, **p < 0.01, ***p < 0.001.

To assess the model’s performance on unseen data, a leave-one-out cross-validation was performed. Our model yielded a mean absolute error (MAE) of 6.32/92 points, a root mean squared error (RMSE) of 8.12/92 points, and retained 37% of the explanatory power, as depicted in Fig. 2B. An illustrative example of the model’s application and performance, using speech data from a participant of this study, is depicted in supplementary video 1.

Further, we examined the model’s predictive capacity for individual MDS-UPDRS-III subscores. Our model demonstrated heightened accuracy in forecasting scores related to bradykinesia (Fig. 2C, r^2^ = 0.35) and axial symptoms (Fig. 2D, r^2^ = 0.33), while showing limited efficacy in predicting rigidity (Fig. 2E, r^2^ = −0.16) and tremor subscores (Fig. 2F, r^2^ = −0.20).

### Digital speech biomarkers to index hyperkinetic symptoms

**Fig. 3:**
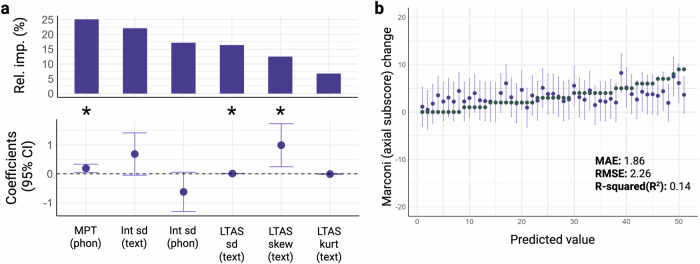
Hyperkinetic model for digital speech features selection and accuracy to detect changes in hyperkinetic symptoms of PD. **a** Regression coefficients, 95% Confidence Intervals, and Relative importances plots from the backward stepwise linear regression model to predict changes in hyperkinetic symptoms of PD (Marconi dyskinesia rating scale axial subscore). **b** Actual value of the change in Marconi dyskinesia rating scale axial subscore (grey) vs the predicted value and respective 95% CI (purple). *p < 0.05, **p < 0.01, ***p < 0.001. Int sd Standard deviation of Intensity, MPT Maximal phonation time, LTAS Long-term averaged spectrum, sd Standard deviation, kurt Kurtosis, skew Skewness, CI Confidence Interval, MAE Mean absolute error, RMSE Root mean squared error. Created in https://BioRender.com.

In the third phase of analysis, we focused on identifying digital speech biomarkers that accurately could reflect changes in hyperkinetic symptoms of PD, namely peak-dose dyskinesia. We observed that the variables that better explained the changes in the axial Marconi dyskinesia rating scale (Table 2 and Fig. 3A) were: maximal phonation time (MPT [phon]), standard deviation of intensity during text reading (monoloudness, Int sd [text]), standard deviation of intensity during phonation task (Int sd [phon]), LTAS sd (text), LTAS skewness (text), and LTAS kurtosis (text). Together, these variables accounted for 37% of the observed variability in the dyskinesia score.

To validate the robustness of our model, we used leave-one-out cross-validation. The model demonstrated a MAE of 1.86/12 points and a RMSE of 2.26/12 points. It retained 14% of the explanatory power in predicting the changes in dyskinesia symptoms, as depicted in Fig. 3B.

### Development of composite hypo- and hyperkinetic speech scores

Using the digital acoustic speech variables identified in the previous steps, we constructed two weighted compound scores aimed at capturing motor changes associated with hypokinetic and hyperkinetic symptomatology. These scores were developed to provide a global comprehensive measure of speech features alteration in response to motor fluctuations (Supplementary Table 3).

**Fig. 4:**
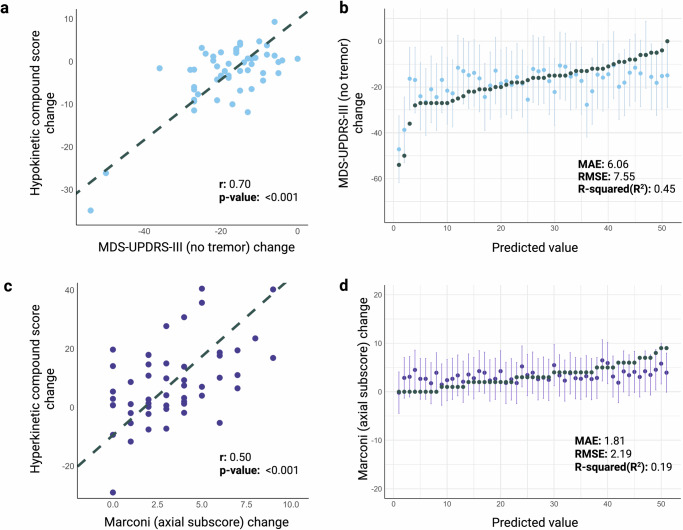
Clinical correlations and predictive accuracy of hypokinetic and hyperkinetic compound scores. **a** Correlation plot between change in the hypokinetic compound score and change in the MDS-UPDRS-III without tremor items. **b** Actual value of the change in MDS-UPDRS-III without tremor items (grey) vs the predicted value using only the hypokinetic compound score and respective 95% CI (blue). **c** Correlation plot between change in the hyperkinetic compound score and change in the Marconi dyskinesia rating scale axial subscore. **d** Actual value of the change in Marconi dyskinesia rating scale axial subscore (grey) vs the predicted value using only the hyperkinetic compound score and respective 95% CI (purple). CI Confidence Interval, MAE Mean absolute error, RMSE Root mean squared error. Created in https://BioRender.com.

We observed a strong positive correlation (Fig. 4A, *r* = 0.70, *p* < 0.001) between changes in the hypokinetic compound score and in the MDS-UPDRS-III (excluding tremor items) across ON and OFF medication states. Moreover, the predictive accuracy of the hypokinetic compound score for changes in MDS-UPDRS-III (no tremor items) was confirmed via LOOCV, as demonstrated in Fig. 4B. For a detailed performance comparison with total MDS-UPDRS-III, please refer to Supplementary Fig. 3. Similarly, changes in the hyperkinetic compound score were strongly positively correlated (Fig. 4C, *r* = 0.50, *p* < 0.001) with modifications in the axial Marconi dyskinesia rating scale. The predictive performance of this score, as determined through LOOCV, closely matched that of the model employing selected individual hyperkinetic features, as shown in Fig. 4D. Importantly, no statistically significant differences were observed in any of the digital speech biomarkers or the compound scores across languages (Supplementary Table 4).

### Use of digital speech biomarkers to predict medication state

In the final phase of our study, we aimed to utilize previously identified digital speech biomarkers of hypokinetic and hyperkinetic symptoms in PD to predict medication state transitions.

**Fig. 5:**
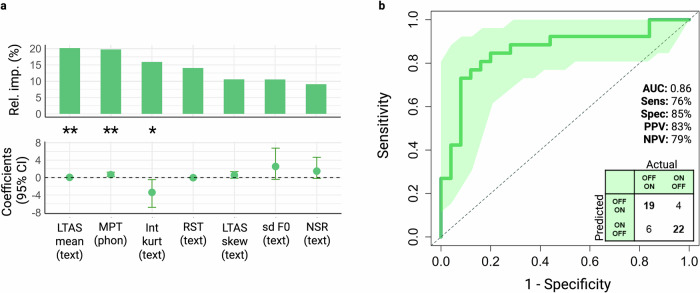
Model for selection of best digital speech biomarkers to predict medication state change and accuracy performance. **a** Regression coefficients, 95% Confidence Intervals, and Relative importances plots from the backward stepwise logistic regression model to predict changes in the medication state in PD patients. **b** Receiver operating characteristic (ROC) curve of the medication state change model and respective performance. *p < 0.05, **p < 0.01. NSR Net speech rate, RST Rate of speech timing, Int Kurt Kurtosis of intensity, sd F0 Standard deviation of fundamental frequency, MPT Maximal phonation time, LTAS Long-term averaged spectrum, skew Skewness, CI Confidence Interval, AUC Area under the curve, Sens Sensitivity, Spec Specificity, PPV Positive predive value, NPV Negative predictive value. Created in https://BioRender.com.

The following biomarkers were selected in descending order of importance: LTAS mean (text), maximum phonation time (MPT [phon]), intensity kurtosis (Int kurt [text]), rate of speech timing (RST [text]), LTAS skewness (text), standard deviation of fundamental frequency (sdF0 [text]), and net speech rate (NSR [text]) (Table 3/Fig. 5A).

**Table 3:** Stepwise backward selection regression model to predict medication state transitions, ordered by relative importance

	Estimate	*p* value
**Medication change Model** (dependent variable: Change OFF to ON or ON to OFF)		
INTERCEPT	−0.34041	0.51172
LTAS mean (text) change	0.04163	<0.01 **
MPT (phon) change	0.66034	<0.01 **
Int kurt (text) change	−3.39710	<0.05 *
RST (text) change	−0.02887	0.05147
LTAS skew (text) change	0.54767	0.14261
sdF0 (text) change	2.54548	0.14421
NSR (text) change	1.48410	0.2086
*Null deviance: 70.681 (50 df); Residual deviance: 29.23; AIC: 45.23*		

*NSR* Net speech rate, *RST* Rate of speech timing, *Int Kur* Kurtosis of Intensity, *sd F0* standard deviation of fundamental frequency, *MPT* Maximal phonation time, *LTAS* Long-term averaged spectrum, *sd* standard deviation, *kurt* kurtosis, *skew* skewness.

*p*-value significance—*p < 0.05, **p < 0.01.

To evaluate the performance of our model, we randomly assigned medication state changes (OFF to ON or ON to OFF) and assessed the model using LOOCV. The model achieved an area under the curve (AUC) of 0.86, accuracy of 80% (95% CI: 0.67–0.90), sensitivity of 76%, specificity of 85%, positive predictive value of 83%, and a negative predictive value of 79%, as can be appreciated in Fig. 5B.

## Discussion

In this study, we identified significant acute effects of dopaminergic medication on speech of PD patients with motor fluctuations. These effects were more pronounced for prosodic, respiratory and averaged spectral features domains. Using a data-driven approach, we identified a set of digital speech biomarkers capable of accurately indexing changes in the hypokinetic and hyperkinetic symptoms of PD induced by dopaminergic medication. Notably, these biomarkers were also effective in detecting medication state changes with high accuracy.

As previously described in the literature, the speech of PD patients in OFF medication condition was significantly slower than healthy controls, which in the present study was captured by the reduced net speech rate and increased duration of pause intervals. These timing abnormalities have been interpreted as consequence of reduced range of orofacial movements and difficulties initiating speech, respectively^24^. In addition, both the slope and standard deviation of the speech intensity during the phonation task were significantly worse in OFF state. This likely arises from the reduced amplitude and impaired control of the respiratory and thyroarytenoid muscles. Monopitch is one of the classical hallmark signs of hypokinetic dysarthria and in the present study, it was significantly more severe in PD patients during OFF state than in healthy controls. Reduced amplitude of vocal cord movements leading to glottal incompetence is usually at the origin of monopitch^23,29^.

Regarding the averaged spectral features of speech, the mean (1^st^ moment), skewness (3^rd^ moment) and kurtosis (4^th^ moment) of PD patients in the OFF-medication condition were significantly different than values of healthy controls. The different moments translate different speech domains but overall represent mainly the reduced vocal cords amplitude and consequent change of the fundamental frequency and glottal incompetence. Globally, all these speech abnormalities observed between PD patients in OFF medication condition and healthy controls reflect the impact of bradykinesia on speech.

During ON medication condition, respiratory function, prosodic abnormalities, and mean of LTAS observed in the OFF-medication condition tended to significantly improve, meaning that the average values changed in the direction of the healthy controls. In contrast, the timing abnormalities observed in OFF worsened in ON medication condition, getting even further apart from the healthy controls reference values, which probably reflects the negative impact of peak-dose dyskinesia on speech. Phenomenologically peak-dose dyskinesia express as chorea^27^, or a combination of chorea and dystonia^27^. Dyskinetic movements affecting axial structures, like the trunk, neck, facial or phonatory system can disrupt speech and hamper speech timing, prosody or articulation domains^29,35^. In our study, although the duration of pause intervals remained mostly unchanged, the net speech rate and rate of speech timing deteriorated in the ON medication condition compared to healthy controls. Similar findings have been also described in other disease models with chorea, such as Huntington’s disease^29,35^. Additionally the differences observed on mean, kurtosis and skewness of LTAS between PD patients in OFF medication and healthy controls, became nonsignificant after levodopa intake. The latter supports the overall normalization of the different spectral moments with dopaminergic medication.

Importantly, we also demonstrated that the overall speech improvement from the OFF to ON state was perceptible using the MDS-UPDRS-III speech item. However, it is clinically challenging to accurately perceive and differentiate changes across the multiple affected speech domains. As also observed in our study, the perceptual speech evaluation using the MDS-UPDRS-III speech item (Supplementary Fig. 4) was most strongly correlated with changes in voice quality (as captured by harmonics-to-noise ratio, HNR). This, combined with the requirement for highly trained personnel to reliably rate speech, may explain why previous studies relying exclusively on perceptual assessments have failed to detect acute changes across medication states in Parkinson’s disease. These findings further support the possible superior granularity and sensitivity of digital speech biomarkers over traditional perceptual evaluations, especially for capturing subtle variations beyond voice quality^1,23^.

The terms akinesia, hypokinesia, and bradykinesia are often used interchangeably to describe the motor disturbances characteristic of Parkinson’s disease (PD)^36^. However, these terms represent distinct phenomena: akinesia refers to delays in initiating voluntary movement, hypokinesia denotes reduced movement amplitude, and bradykinesia describes the slowing of movements already underway^10,37^. In this study, we use the term “hypokinetic” broadly to encompass these three aspects of motor impairment typical of PD. In the next paragraphs, we further explore and disentangle the differences between these phenomena and hypothesize how they manifest in distinct ways across various digital speech biomarkers.

Among the eight digital speech biomarkers indicating hypokinetic features of the disease, the most contributory biomarkers selected in our model were prosody variables such as monopitch (sd F0) and monoloudness (sd Int and Int kurt), which are two of the prototypical abnormalities observed in hypokinetic dysarthria^23^. Both these variables denote the reduction of amplitude of vocal cords, thyroarytenoid and respiratory muscles movements, which were strongly correlated with changes in bradykinesia subscore in our study, particularly the monopitch (Supplementary Fig. 4). We interpret these speech abnormalities as specific markers of amplitude reduction, closely capturing the hypokinesia phenomena characteristic of Parkinson’s disease. However, the standard deviation of intensity was also important to predict axial dyskinesia. This dual role in both hypokinetic and hyperkinetic symptoms align with our understanding of the disease’s impact. Specifically, the disease typically reduces the variability of speech volume, resulting in monoloudness. Consequently, improvements in motor symptoms are expected to restore some of this variability. However, dyskinesia can induce abrupt and irregular movements of the vocal cords and respiratory muscles. This can lead to considerable fluctuations in speech loudness, thereby increasing the variability of intensity.

Speech timing variables such as rate of speech timing and net speech rate were also relevant to explain motor changes. Globally we interpret the changes in these timing variables as proxy of the bradykinesia component of the disease^38^. In the context of speech, bradykinesia can be characterized by a noticeable slowing of speech production and diminished articulatory precision, resulting in overall lower values of net speech rate and rate of speech timing as confirmed in our study^23,24,39,40^. However, it is important to keep in mind that timing variables in PD do not always behave in a homogeneous way. Besides slowness of speech due to bradykinesia^23,24^, sudden increase in speech rate^41,42^ due to festination have also been described in PD.

The mean and skewness of LTAS, were also relevant to predict hypokinetic symptoms of PD. The overall spectral mean reflects mainly changes in the fundamental frequency, which is typically decreased in PD as a consequence of the reduced amplitude of vocal cord movements^43^. On the other hand, spectral skewness is indicative of glottal closure during phonation, with reduced skewness associated with hyperadduction and increased skewness associated with hypoadduction^43^. Reduced amplitude of vocal cords typical of hypokinesia, normally translates as reduced skewness, whereas dyskinetic involuntary movements could produce glottal hypoadduction, thus justifying the opposite effects observed on this variable in the hypokinetic and hyperkinetic models.

Voice to onset time was the last variable selected in the hypokinetic model. This variable, related to consonant articulation, reflects the slowness in initiating lip and tongue movements, a hallmark of akinesia in Parkinson’s disease^44–46^.

In turn, hyperkinetic symptoms were mostly explained in our study by speech variables from respiratory and loudness variability. Respiratory capacity was explored in our study with the maximal phonation time, and we found that patients in ON medication condition with dyskinesia were more likely to hold longer phonations in comparison with OFF medication condition. A possible interpretation is that this increase is caused by the improved ventilatory ability during ON medication condition. Accordingly, this variable could have been retained in our model of dyskinesia because dyskinetic PD patients might be the ones with better dopaminergic response. The latter implies that dyskinetic patients not only would have more severe dyskinesia scores, but also a more severe OFF symptomatology and minimal parkinsonian symptoms in ON medication condition. Alternatively, in the phonation task if the air stream is intermittently halted by the dyskinesia, it might be possible that involuntary movements serve as a mechanism that led to a more controlled release of air, ultimately translating into longer phonation times. In support of the last hypothesis is the fact that standard deviation of intensity during phonation was smaller in ON medication condition.

Loudness variability has been shown to be highly associated with choreiform movements in Huntington’s disease^35^. Similarly, in our study speech variables indexing average loudness variability such as standard deviation of intensity during phonation and reading task, had a particularly important role predicting dyskinesia.

Different spectral moments such as standard deviation, skewness and kurtosis of LTAS were also important to predict changes in dyskinesia score. Spectral features are harder to interpret but the positive association of changes in skewness and standard deviation of LTAS and changes in dyskinetic score likely arises from the dyskinesia of the vocal cords. This can result in sudden, excessive abduction of the vocal cords and increased variability in the resonant components of the entire speech apparatus, due to dyskinetic movements of all the articulatory and respiratory muscles involved.

Importantly, utilizing the identified hypokinetic and hyperkinetic speech features it was possible to create two compound scores (hypokinetic and hyperkinetic compound scores), which demonstrated a similarly good performance in predicting the hypokinetic and hyperkinetic outcomes. This capability is particularly important for future clinical trials, as it allows for the use of simple speech recordings as an outcome measure to objectively monitor both hypokinetic and hyperkinetic features of PD. Such biomarkers could also be precious in future to guide closed loop treatment adaptation.

Starting from a pool of speech biomarkers found to accurately index hypokinetic and hyperkinetic motor symptoms of PD, we successfully developed a model to predict modifications of the medication state. Symptoms of PD are known to be very heterogenous and vary significantly from person to person^9^. Hence, it is not surprising to see significant interindividual variability among the different digital speech biomarkers^23^. Therefore, when predicting speech modulation across different medication states, it is crucial to consider the unique speech characteristics or “fingerprint” of each individual. Analyzing individual speech changes between medication states, rather than relying solely on group-level predictions of ON and OFF states, enhances the accuracy and personalization of these predictions.

With our model we obtained a very good accuracy, with an area under the curve of 0.86. The most important digital speech biomarkers to predict medication-state transitions were a mixture of the most salient biomarkers to predict hypokinetic symptoms such as standard deviation of fundamental frequency, kurtosis of intensity, speech timing variables (RST and NSR), and mean of LTAS, as well as variables also related with hyperkinetic features prediction, like LTAS skewness and maximal phonation time (MPT). The fact that our model retained variables from both hypokinetic and hyperkinetic models, highlights the importance of both phenomena to predict the medication transitions as well as reinforces the strength of our motor speech models.

Recently, Norel R., et al.^47^ demonstrated the feasibility of detecting medication states in a small cohort of PD patients, achieving high precision (0.89, 0.84, and 0.60 accordingly to the speech task used). Their approach relied on variables such as Mel frequency cepstral coefficients (MFCCs) and semantic content, which are less directly linked to physiological motor functions and achieved higher accuracy only in cognitively demanding tasks. Additionally, their results might have been overinterpreted due to the lack of systematic validation using a control group. Our method utilizes a standardized motor speech protocol supported by robust literature^29^, proven stability across languages^2^, and minimal cognitive demand. This protocol, which takes only 5–10 minutes to administer, is well-suited for broader PD populations and accurately reflects motor symptoms. Moreover, we showed that repeating the protocol under identical conditions (repeated medication OFF assessments) yielded consistent results, underscoring its stability and reliability.

Notably, assessing speech in PD presents unique challenges, as traditional perceptual assessments can be biased towards the dominant effect of voice quality. While these methods are valuable, they might not fully capture the subtle and complex changes in PD speech. Our study demonstrates that voice quality alone does not encompass the full range of PD speech changes, highlighting the potential of digital speech biomarkers to provide a more comprehensive analysis.

Additionally, current motor clinical assessments in PD such as MDS-UPDRS-III, are labor and time demanding, and suffer from significant inter- and intra-rater variability. Even among senior movement disorders specialists, the inter-rater variability in UPDRS-III can reach as high as 12.5/108 points, while the intra-rater variabilities can be as high as 8/108 points^48^. In contrast, our proposed speech assessment is rapid, minimally dependent on the examiner, and provides a more objective and reliable measurement, with a mean absolute error of 6.32 in predicting hypokinetic symptoms of PD. This attribute enhances its value for clinical research, offering the potential to be scalable to large populations, where it can be used as a valuable safety and/or efficacy outcome measure for surgical or pharmacological interventions.

Objective speech monitoring also holds promise to be easily transposed outside the clinical setting^49^. An expected application, is the use of speech recordings to assess the fluctuations of the disease at home using common devices such as smartphones or smartwatches^50,51^. A tool capable of dissociating the beneficial effects of levodopa on akinesia-related speech from its detrimental effects due to dyskinesia would, in our view, offer substantial clinical value. This distinction may be particularly relevant in future clinical trials, where simple speech recordings could serve as outcome measures to objectively capture both hypokinetic and hyperkinetic features of Parkinson’s disease. Moreover, such digital biomarkers could eventually enable the development of speech-based, adaptive closed-loop therapeutic strategies^69^.

However, there are currently some challenges to use this technology in a noncontrolled scenario^49,52^. Differences in environmental noise, microphone quality or microphone positions can hinder the use of digital speech biomarkers. In particular, amplitude measures such as intensity are more vulnerable while frequency measures such as pitch appear to be more robust against these confounding factors^53^.

Currently, several strategies have demonstrated the feasibility of integrating speech assessments into daily life of PD patients^49^. One approach involves passive recording of phone calls, which requires minimal patient effort and has shown promise in distinguishing PD and prodromal PD patients from healthy controls^51,54^. Another approach involves web-based platforms or apps guiding patients through a set of active speech tasks, which have demonstrated possible superior discrimination power over standard clinical scales in clinical trials assessing disease progression^55^.

Importantly, it is critical to ensure legal and ethical frameworks, where individual patient data can be securely processed, analyzed and not used by other parties for different purposes as the ones explicitly consented by the patients^56^.

A major strength of our study lies in the use of a comprehensive set of well-validated digital speech biomarkers, combined with rigorous clinical assessments and robust validation methods. Additionally, the linguistic diversity in Switzerland, enabled us to investigate digital speech biomarkers across 4 different languages. This enhances the stability and generalizability of our findings, aligning with previous multicentric studies that demonstrated the robustness of similar digital speech biomarkers across five different languages^2^.

Nevertheless, several limitations should be acknowledged. First, the study population consisted of individuals with moderate to advanced Parkinson’s disease and prominent motor fluctuations, evaluated under a suprathreshold levodopa challenge. While this design allows for a strong within-subject contrast of medication effects, further research is required to validate these findings across a broader range of disease stages and medication doses. Second, although healthy controls served primarily as a reference for digital speech biomarker ranges, their average age was slightly higher than that of the PD cohort. While this difference was statistically controlled using multivariate regression models, some residual confounding cannot be excluded. However, since speech typically deteriorates with age, more closely age-matched controls may in fact strengthen the group-level contrasts found. Finally, we acknowledge that future studies, preferably based on larger sample size and multicentric design, should further replicate and extend our findings.

In conclusion, our study demonstrated that digital speech biomarkers can effectively index changes in both hypokinetic and hyperkinetic motor symptoms of PD. These biomarkers can accurately predict transitions in dopaminergic medication states at an individual level. Therefore, we believe the results of this study supports the integration of modern digital speech analysis into clinical practice and research. The composite speech scores developed in this study are particularly relevant for clinical research, where they can serve as reliable motor outcomes or safety measures. Additionally, by passively monitoring motor symptoms, the motor speech biomarkers identified in this study can empower patients with PD and their neurologists with more detailed and granular data. This could in future facilitate more personalized and responsive treatment adjustments, ultimately enhancing the quality of life of individuals suffering from PD.

## Methods

### Study design and participants

A total of 55 consecutive patients that underwent a levodopa challenge at Inselspital, Bern, Switzerland, between 2021 and 2023, as part of the routine assessment for advanced therapies of PD were reviewed. In this clinical observational study, patients that fulfilled the diagnosis of PD, according to Movement Disorders Society (MDS) diagnosis criteria^57^, completed the speech examination during the levodopa challenge, and signed the general consent for biomedical research were included (Supplementary Fig. 1). Four patients were excluded after the levodopa test due to diagnosis revision (*n* = 2), absence of signed informed consent for biomedical research (*n* = 1) or insufficient quality of the speech recording (*n* = 1).

All patients were assessed in a practically defined OFF medication state ( ≥ 8 h after last levodopa intake on the previous day and/or ≥ 48 h dopamine agonist withdrawal), and then were retested in ON medication condition (30–60 min after the administration of a fast-acting soluble formulation of levodopa/benserazide 100/25 mg equivalent to 150% of patient’s usual levodopa equivalent morning dose). Levodopa equivalent doses were calculated according to previously described conversion factors^58^.

To assess the statistical power of our analyses based on the PD dataset, we conducted a power analysis using the pwr package in R. A previous study employing a similar study design and digital speech biomarkers, assessing patients in two medication conditions (med OFF vs. med ON) but at earlier disease stages, reported an effect size of Cohen’s d = 0.5^24^. Given a paired t-test design, a sample of 51 patients evaluated in both ON and OFF states, and a significance level (α) of 0.05, our study was powered to 0.94 for detecting a moderate effect size (d = 0.5) and 0.998 for a large effect size (d = 0.7). Moreover, to ensure that the results reported were not the consequence of simple speech variability or due to a learning effect of the speech tasks, the speech of a sub-group of 10 PD patients was assessed three times. Two times in the practically OFF medication condition with an interval of 15 minutes, and one time in the practically defined ON condition, 30–60 min after the intake of levodopa.

Forty-three healthy controls matched for language, gender, and age—as much as possible—from a previous multicentric study using equivalent speech recording examination were included. The healthy controls served as reference for the magnitude of changes observed in the patients with PD^2^.

The retrospective analysis of data from patients, who provided general consent for biomedical research, was approved by the local ethics committee (KEK 2023-01427) and conducted in accordance with the ethical standards established in the Declaration of Helsinki and its subsequent amendments^59^.

### Neurological examination

The severity of the hypokinetic motor symptoms of PD was quantified using the Movement Disorder Society - Unified Parkinson’s Disease Rating Scale (MDS-UPDRS) part III, excluding tremor items^60^. The option to exclude the tremor items was taken because phenomenologically it is considered a hyperkinetic sign. Moreover, tremor in PD is classically more severe on the limbs while the patient is at rest and can be disrupted by voluntary actions^38^. Since speech is an active task, its inclusion would mostly introduce noise in our outcome measure. Additionally, tremor is absent in about 25% of the patients^61^, while bradykinesia is the core symptom required for the diagnosis of PD, and henceforth we restrict our analysis to the hypokinetic symptoms of the disease.

Subscores of the cardinal features of PD were derived by the sum of the relevant items from the MDS-UPDRS-III as follows: bradykinesia (items 3.4–3.8), rigidity (items 3.3), axial (items 3.1–3.2, 3.9–14), and tremor (items 3.15–18). Severity of motor symptoms was assessed twice (in OFF and ON medication conditions) by experienced clinical staff.

Marconi Dyskinesia rating scale was used in each medication condition to rate the presence and severity of dyskinesia^26^. An axial subscore of the Marconi dyskinesia rating scale was derived from the face, neck and trunk items, since the impact of limb dyskinesia on speech was considered negligible. The remaining scales were filled out only in ON medication condition since they refer to severity of different symptoms over the past weeks. Overall cognition was quantified using the Montreal Cognitive Assessment (MoCA)^62^. The non-motor aspects of the disease were estimated with the MDS-UPDRS part I, experiences of daily living with MDS-UPDRS part II^63^. Severity of motor complications was measured with MDS-UPDRS part IV^63^.

### Speech examination

Recordings were performed in a quiet room with low ambient noise level. To ensure good quality of the speech recordings, a head mounted condenser microphone was used (Shure Beta 53; Shure, Niles, IL, USA), installed with a distance between microphone and mouth of around 5 cm and then connected to a recording device (TASCAM DR-40). Speech signals were sampled at 48 kHz with 16-bit resolution. All participants were asked to perform three speaking tasks: (i) sustained phonation (phon) of the vowel /a/ per one breath for as long and steadily as possible, (ii) fast repetition of syllables /pa/-/ta/-/ka/ (ddk) twelve times per one breath as precise and fast as possible, and (iii) a standardized phonetically balanced reading passage (text) composed of > 100 words (ranging from 113 to 151 words, depending on the language). The first 2 tasks were performed twice to ensure greater stability, whereas the third task was performed just once per examination^29^. The three speaking tasks were selected because they can provide the necessary information for the accurate description and interpretation of motor speech disorders in PD^29^.

### Speech analysis

Based on previous studies assessing different types of hypokinetic^2,23,24,64–66^ and hyperkinetic^35,67–69^ dysarthria, 12 basic digital speech biomarkers, measuring different perceptual domains of hypokinetic and hyperkinetic dysarthria described in the landmark study of Darley et al.^4^, and 4 moments of the long-term averaged spectrum (LTAS) were selected to reduce the probability of type I errors and collinearity.

Briefly, monopitch was measured by the standard deviation of fundamental frequency (sd F0) during the reading task, while monoloudness by measures of speech intensity variability (standard deviation [Int sd] and kurtosis [Int kurt]) during the reading text task. Imprecise consonants can be measured using voice to onset time (VOT) in the fast repetition of syllables task. Dysphonia, perceptually understood as harsh/breathy voice, was estimated via harmonics-to-noise ratio (HNR) during the phonation task. To understand patient’s respiratory speech function, maximal phonation time (MPT) and exploratorily slope of intensity (Int slope) during phonation task were analyzed. Speech timing abnormalities were estimated by measures like net speech rate (NSR), duration of pauses intervals (DPI), and the rate of speech timing (RST) during the reading text task. To investigate the hyperkinetic component of speech, the variability of the intensity during phonation task (Int sd) and the skewness of intensity (Int skew) during the reading text were added as exploratory variables.

Additionally, different LTAS moments were included. Specifically, the LTAS mean (first moment) has been correlated with alterations in vocal loudness and fundamental frequency. The standard deviation (second moment) serves as an indicator of the vocal tract’s resonant properties, which are often modified in PD and may elude detection by basic speech biomarkers. Furthermore, skewness (third moment) is associated with anomalies in glottal closure, whereas kurtosis (fourth moment) reflects disturbances in glottal control mechanisms^43,70^. A more detailed description and interpretation of the 16 speech features used are provided in Supplementary Table 1.

All signal processing and speech analysis steps were done in MATLAB (MathWorks, Natick, MA). Comprehensive algorithmic details, packages used and accuracy on individual acoustics features^1,66,71,72^ and LTAS moments^43^ have been previously reported.

### Statistical analysis

To evaluate the distribution patterns of each digital speech biomarker, we initially inspected the distributions via histograms and used the Shapiro-Wilk test to assess and confirm normality. Depending on the data distribution, comparisons between the OFF and ON medication states for each biomarker were conducted using paired t-tests (for normally distributed data) or Wilcoxon Signed-Rank tests (for non-normally distributed data). Similarly, to investigate differences in speech features across PD patients (medication ON and OFF conditions) and healthy controls, we employed a series of multivariable linear regression models. Each speech feature was treated as a dependent variable in an individual regression model, allowing us to systematically evaluate the group effects while adjusting for age, gender and global cognition (MoCA score). The Holm-Bonferroni method was applied to adjust for multiple comparisons. Effect sizes were calculated using Cohen’s d for paired t-tests, rank-biserial correlation for non-parametric tests, and standardized beta (ß) coefficients for regression models. Thresholds for interpretation were ≤0.2 for small, ≤0.5 for medium, and ≥0.8 for large effects.

Backward stepwise regression was employed to identify digital speech biomarkers that better explained the variability in hypokinetic and hyperkinetic symptoms of the disease accordingly to the Akaike Information Criterion (AIC). For the hypokinetic model, changes in the MDS-UPDRS-III excluding tremor items, between medication ON and OFF states served as the dependent variable. Conversely, for the hyperkinetic model, the dependent variable was the change in axial Marconi dyskinesia rating scale between the two medication states. To assess the contribution of each digital speech biomarker within the backward stepwise linear regression models, we computed the relative importance metric, proportional marginal variance decomposition (pmvd), as proposed by Feldman in 2005^73^.

After identifying the set of digital speech biomarkers that better explain the changes in hypokinetic and hyperkinetic features of PD, each speech parameter was standardized (z-scored). The z-scored digital speech biomarkers were then weighted according to their relative importance derived from the backward stepwise regression models. As a final step, a hypokinetic compound score was computed by aggregating all selected weighted z-scored speech features, adhering to the directionality of the coefficients obtained from the hypokinetic regression model. Similarly, a hyperkinetic compound score was generated using the same methodological approach, tailored to the characteristics and coefficients of the hyperkinetic stepwise backward regression model.

To evaluate the robustness of the final backward stepwise regression models, we implemented leave-one-out cross-validation (LOOCV). The predictive accuracy of each model was quantified using the mean absolute error (MAE), root mean squared error (RMSE), and R-squared values.

Lastly, to investigate the ability of individual speech biomarkers to predict medication state transitions, we designed a methodology to simulate a real-world scenario. Recognizing the heterogeneity of speech abnormalities in PD—where different patients may exhibit varying degrees of impairment across distinct speech domains—we focused on changes in individual biomarkers rather than directly predicting medication states (OFF vs. ON). This approach better capture the individualized impact of medication. To evaluate the predictive accuracy of digital speech biomarkers in detecting medication state transitions (OFF to ON or ON to OFF), each subject was randomly assigned a binary value (0 or 1) with equal probability. For subjects assigned 0, the absolute difference between OFF and ON values for each speech biomarker was computed to represent an OFF-to-ON transition. For subjects assigned 1, the difference was calculated by subtracting the OFF from the ON value, simulating the ON-to-OFF transition. A stepwise backward logistic regression model was applied to the previously selected hypokinetic and hyperkinetic digital speech features to identify the most useful predictors of medication state transitions. The model’s performance was evaluated using LOOCV. Metrics such as model accuracy, sensitivity, specificity, positive and negative predictive values, and the area under the receiver operating characteristic curve (AUC) were calculated.

All statistical analyses and graphical representations were conducted using R software, version 4.4.0, dated 2024-04-24.

## Supplementary information

Supplementary information

Supplementary video 1

## Data Availability

The extracted data supporting the findings of this study are available upon request from the corresponding author. The voice recordings are not available due to participant’s privacy concerns and the sensitive nature of the data.
