# Supplementary material for: Digital speech biomarkers can measure acute effects of levodopa in Parkinson’s disease
Source: NPJ Parkinsons Dis. 2025 Jul 1;11:184. doi: 10.1038/s41531-025-01045-5 (PMC12217330; doi:10.1038/s41531-025-01045-5)
Supplement: Supplementary file 1 — Supplementary information [file 41531_2025_1045_MOESM1_ESM.pdf]

SUPPLEMENTARY MATERIAL

TABLE OF CONTENTS

Supplementary Tables

Supplementary Table 1. Overview of applied acoustic measures and its proposed interpretation. 3

Supplementary Table 2. Baseline and clinical characteristics of the subgroup of PD patients that underwent 2 OFF plus 1 ON medication recording 5

Supplementary Table 3. Linear regression model to predict changes in hypokinetic and hyperkinetic symptoms of PD using the speech hypokinetic and hyperkinetic compound score 6

Supplementary table 4. Mean and standard deviation comparisons of the changes between medication ON and OFF in each speech features across languages 7

Supplementary Table 5. Statistical tests, p-values and effect size of individual digital speech biomarkers significantly different in PD patients across medication conditions (ON vs. OFF), between healthy controls and PD patients in OFF medication, and heathy controls and PD patients in ON medication condition 8

Supplementary Figures

Supplementary Figure 1. Detailed flowchart of patient's recruitment 9

Supplementary Figure 2. Consistency of Individual Digital Speech Biomarkers Across Repeated OFF Medication Recordings. 10

Supplementary Figure 3. Accuracy to predict the total MDS-UPDRS-III score using the selected hypokinetic digital speech biomarkers, the hypokinetic compound speech score and correlation of these predictions with clinical score 11

Supplementary Figure 4. Correlation Analysis of Clinical Variables and individual digital speech biomarkers 12

Video Captions

Supplementary Video 1. Application of automated speech analysis in detecting speech abnormalities and predicting overall hypokinetic motor symptom changes 13

Supplementary Tables

Supplementary Table 1. Overview of applied acoustic measures and its proposed interpretation.

	Deviant speech Dimension/LTAS measure (speaking task)	Acoustic feature/LTAS moment	Definition	Pathophysiological interpretation
**Voice Quality**	Harsh voice (sustained phonation)	HNR (Harmonics-to-noise ratio)	amount of noise in the speech signal.	HYPO (↓): Reduced rate of airflow and improper control of vocal folds causes increased turbulent noise. HYPER(↓):Reduced rate of airflow and improper control of vocal folds caused by dyskinesia may cause turbulent noise.
**Articulation deficits**	Imprecise consonants (syllable repetition)	VOT (Voice onset time)	length of the entire consonant from initial burst to vowel onset.	HYPO (↑): Slowness of lip and tongue movements, leads to a longer time required to pronounce individual consonants. HYPER (↑): Dyskinesia may impair lip and tongue movements coordination leading to a longer time required to pronounce individual consonants.
**Timing abnormalities**	Articulation rate (reading passage)	NSR (Net speech rate)	total number of syllables divided by the total duration of speech after removal of pauses.	HYPO (↓ or ↑): Impaired control of orofacial muscles leads to a decrease in speech rate. Speech festination can increase the speech rate. HYPER (↓ or ↑): dyskinesia can decrease or increase the speech rate accordingly to muscles and time the involuntary movements appear.
	Quality of speech timing (reading passage)	RST (Rate of speech timing)	Rate of voiced, unvoiced and pause intervals measured as the slope of the regression line of total interval count per time.	HYPO (↓ or ↑): reduced stream of voiced, unvoiced and pause intervals. Typically, in consequence to reduced range of speech movements and/or decreased syllabic rate. Speech festination can increase the speech rate. HYPER (↓ or ↑): dyskinesia can lead to increased pauses and reduction of voiced intervals, reducing the rate of speech or shorten the pause intervals and unvoiced intervals, consequently increasing the rate of speech.
	Inappropriate silences (reading passage)	DPI (Duration of pause intervals)	the median length of pause intervals.	HYPO (↑): makes initiating speech difficult, leading to prolonged pause intervals. HYPER (↑): Dyskinesia may difficult initiation of speech and inappropriate timing leading to prolonged pause intervals.
**Prosody abnormalities**	Monoloudness (reading passage)	Int SD (Sd of Intensity)	Standard deviation of speech intensity contour extracted from voiced segments.	HYPO (↓): decreases amplitude of respiratory and thyroarytenoid muscles. HYPER (↑):Inappropriate coordination of speech organs leading to unstable loudness of individual syllables.
	Intensity kurtosis (reading passage)	Int Kur (Kurtosis of intensity)	Kurtosis of speech intensity contour extracted from voiced segments.	HYPO (↓?): decreased amplitude of respiratory and thyroarytenoid muscles may lead to uniformity of speech loudness. HYPER (↑?): hypothesized that sporadic bursts of loudness caused by deficient motor control may cause increased kurtosis.
	Intensity skewness (reading passage)	Int skew (Skewness of intensity)	Skewness of speech intensity contour extracted from voiced segments.	HYPER (↑ or ↓): hypothesized that due to bursts of louder speech amidst generally softer speech production, could lead to a positive skewness in speech intensity. If loudness is good, sudden interruption by dyskinesia may conversely produce sudden low intensity moments.
	Monopitch (reading passage)	F0 SD (Sd of fundamental frequency)	Standard deviation of the contour of fundamental frequency converted to semitone scale.	HYPO (↓): reduced amplitude of vocal cord movements, leading to glottal incompetence. HYPER (↓): dyskinesia may interrupt vocal cord movements which leading to glottal incompetence.
**Respiratory**	Respiratory control (sustained phonation)	MPT (Maximal phonation time)	the total duration of all voiced intervals detected by segmentation.	HYPO (↓): causes laryngeal weakness and weakness of the respiratory musculature. HYPER (↓): brisky dyskinesia on axial muscles may cause premature interruption of phonation.

	Intensity variability (sustained phonation)	Int SD (Sd of Intensity)	Standard deviation of speech intensity contour extracted from the sustained phonation task.	HYPO (↑): decreased amplitude of respiratory and thyroarytenoid muscles leads to quick reduction of intensity.<br>HYPER (↑): Involuntary movements can lead to intensity variability during phonation task.
	Respiratory resistance (sustained phonation)	Int slope (slope of intensity)	Regression slope of speech intensity contour extracted from phonation task.	HYPO (↓): reduced vocal and respiratory muscles control, leads to steeper declines of intensity.
**LTAS moments**	LTAS mean (reading passage)	1st LTAS moment	The spectral mean or spectral centroid.	HYPO (↓): reduced amplitude of vocal cord movements, leads to reduction in the fundamental frequency of the voice and reduction of overall spectral mean.<br>HYPER (?): exploratory
	LTAS sd (reading passage)	2nd LTAS moment	The spectral standard deviation.	HYPO (↓): reduced amplitude of vocal cord movements, leads to reduction in variability of the fundamental frequency of the voice and reduction of overall spectral standard sd.<br>HYPER (?): exploratory
	LTAS skewness (reading passage)	3rd LTAS moment	Measure of the asymmetry of the frequency distribution of energy.	HYPO (↑): glottal hypoadduction together with glottal incompetence leads the spectral distribution to be positively skewed in PD.<br>HYPER (?): exploratory
	LTAS kurtosis (reading passage)	4th LTAS moment	Measure of outliers in the energy distribution.	HYPO (↑): reduced amplitude of vocal cord movements and impaired control leads to LTAS uniformity which translates into increased kurtosis.<br>HYPER (?): exploratory

Abbreviations: sd – standard deviation; LTAS – Long-term Averaged Spectrum; HYPO – hypokinetic diseases; HYPER – hyperkinetic diseases/dyskinesia;

Supplementary Table 2. Baseline and clinical characteristics of the subgroup of PD patients that underwent 2 OFF plus 1 ON medication recording.

	**PD (n=10)**
**Age (years), mean (sd)**	65.1 (4.68)
**Gender m/f (%)**	7/3 (70/30)
**Language (%)**	
- **German**	9 (90)
- **French**	1 (10)
**Disease duration (years) mean (sd)**	9.2 (3.26)
**MoCA, mean (sd)**	25.3 (4.69)
**LEDD (mg/d), mean (sd)**	1137.0 (396.12)
**Levodopa dose given (mg), mean (sd)**	278.0 (57.31)
**MDS-UPDRS I, mean (sd)**	11.0 (4.57)
**MDS-UPDRS II, mean (sd)**	14.3 (6.57)
**MDS-UPDRS III OFF, mean (sd)**	46.0 (11.27)
**MDS-UPDRS III ON, mean (sd)**	21.4 (9.56)
**MDS-UPDRS IV, mean (sd)**	8.6 (4.6)
**Marconi dyskinesia scale OFF, mean (sd)**	0 (0)
**Marconi dyskinesia scale ON, mean (sd)**	6.2 (6.0)

Abbreviations: PD - Parkinson’s disease; HC – Healthy Controls; sd – standard deviation; MoCA – Montreal Cognitive Assessment; LEDD – Levodopa equivalent daily dose; MDS-UPDRS-III - Movement Disorder Society-Unified Parkinson's Disease Rating Scale part 3; H&Y – Hoehn and Yahr; n.a. – not-available; pts – points; m – male; f – female

Supplementary Table 3. Linear regression model to predict changes in hypokinetic and hyperkinetic symptoms of PD using the speech hypokinetic and hyperkinetic compound score.

	Estimate	p value
**HYPOKINETIC COMPOUND SCORE MODEL** (dep. var.: MDS-UPDRS-III (no tremor) change)		
INTERCEPT	-15.1985	<0.001 ***
Hypokinetic compound score	0.9963	<0.001 ***
Res. std. error: 7.403 (49 df); Multiple R2: 0.49; Adj. R^2*: 0.48; F-statistic: 48.01; p-value: <0.001*		
**HYPERKINETIC COMPOUND SCORE MODEL** (dep. var.: Marconi (axial subscore) change)		
INTERCEPT	2.52492	<0.001 ***
Hyperkinetic compound score	0.09233	<0.001 ***
Res. std. error: 2.156 (49 df); Multiple R2: 0.25; Adj. R^2*: 0.23; F-statistic: 16.0; p-value: <0.001*		

Abbreviations: p-value significance: *p<0.05, **p<0.01, ***p<0.001, p<0.1

Supplementary table 4. Mean and standard deviation comparisons of the changes between medication ON and OFF in each speech features across languages

Digital speech biomarker change	DE n=34	FR n=11	IT n=5	K-W test	p value	EN† n=1
**HNR (phonationA)**	0.6 (2.4)	0.5 (1.3)	1.4 (2.0)	0.83	0.66	2.4
**MPT (phonationA)**	2.4 (5.0)	1.4 (3.3)	2.9 (3.5)	0.54	0.76	-1.6
**IntStd (phon)**	-0.3 (0.9)	-0.4 (1.1)	-0.6 (0.4)	2.29	0.32	-0.5
**IntSlope (phon)**	0.2 (0.5)	0.2 (0.4)	0.2 (0.2)	1.25	0.54	0.01
**VOT (ddk)**	-1.4 (5.6)	-0.9 (2.0)	-2.5 (5.1)	0.11	0.95	-1.5
**RST (text)**	-23.0 (59.7)	-34.3 (78.4)	-20.1 (91.4)	0.05	0.97	12.0
**DPI (text)**	-0.5 (46.9)	34.7 (99.1)	4.5 (47.4)	0.09	0.96	-21.8
**stdPWR (text)**	0.2 (0.8)	0.4 (1.3)	0.4 (0.8)	0.29	0.87	1.3
**stdF0 (text)**	0.3 (0.5)	0.2 (0.6)	-0.02 (0.7)	5.07	0.08	0.04
**NSR (text)**	-0.04 (0.4)	0.05 (1.6)	-0.3 (0.4)	1.53	0.47	-0.3
**IntSkew (text)**	0.01 (0.1)	0.05 (0.2)	0.05 (0.1)	1.85	0.40	0.1
**IntKurt (text)**	0.1 (0.3)	-0.14 (0.7)	-0.17 (0.3)	5.59	0.06	-0.4
**LTASmean (text)**	28.7 (50.6)	26.2 (57.6)	13.0 (35.3)	0.50	0.78	55.4
**LTASstd (text)**	49.6 (162.4)	103.5 (175.5)	47.0 (139.0)	0.54	0.76	32.8
**LTASskew (text)**	-0.6 (2.2)	-1.1 (2.7)	-1.0 (2.5)	0.23	0.89	-1.9
**LTASkurt (text)**	-20.2 (111.1)	-46.2 (131.3)	-49.3 (127.3)	0.31	0.86	-42.0
**Hypokinetic compound score**	-2.8 (6.9)	-3.0 (9.3)	-1.7 (6.2)	0.16	0.92	-9.0
**Hyperkinetic compound score**	6.9 (12.4)	9.8 (17.4)	12.2 (10.6)	0.86	0.65	10.8

Abbreviations: K-W test: Kruskal-Wallis test; ° p<0.1 and p>0.05; † - Please note that the English spoken group only contained 1 patient. This column contains only the individual values of this patient for reference but was not included in the group comparison statistic.

Supplementary Table 5. Statistical tests, p-values and effect size of individual digital speech biomarkers significantly different in PD patients across medication conditions (ON vs. OFF), between healthy controls and PD patients in OFF medication, and heathy controls and PD patients in ON medication condition.

Speech biomarker	PD OFF vs PD ON			PD OFF vs HC†			PD ON vs HC†		
	Statistical test	Corrected p-value	Effect size	Statistical test	Corrected p-value	Effect size	Statistical test	Corrected p-value	Effect size
**VOT (ddk)**	-	-	-	-	-	-	-	-	-
**MPT (phon)**	W=0.002	p=0.06$^{\Delta}$	r=0.44 °°	-4.16	p=0.06$^{\Delta}$	$_{std}$es=0.98	-	-	-
**HNR (phon)**	-	-	-	-	-	-	-	-	-
**RST (text)**	-	-	-	-47.91	p=0.05$^{\Delta}$	$_{std}$es=0.96	ß=-76.50	p<0.001***	$_{std}$es=0.91 °°°
**DPI (text)**	-	-	-	72.25	p<0.001***	$_{std}$es=1.48	ß=-82.05	p<0.001***	$_{std}$es=0.89 °°°
**Int std(text)**	-	-	-	-	-	-	-	-	-
**stdF0 (text)**	W=308.0	p=0.03*	r=0.47 °°°	-0.69	p<0.001***	$_{std}$es=1.61	ß=-0.44	0.05$^{\Delta}$	$_{std}$es=0.66 °°°
**NSR (text)**	-	-	-	-0.83	p<0.001***	$_{std}$es=1.27	ß=-0.88	p<0.001***	$_{std}$es=0.87 °°°
**Int Skew (text)**	-	-	-	-	-	-	-	-	-
**Int Kurt (text)**	-	-	-	-	-	-	-	-	-
**Int std (phon)**	-	-	-	1.12	p<0.001***	$_{std}$es=1.77	ß=0.82	p<0.001***	$_{std}$es=1.01 °°°
**Int slope (phon)**	W=291.0	p=0.02*	r=0.49 °°°	-0.40	p<0.001***	$_{std}$es=1.37	ß=-0.22	p=0.04*	$_{std}$es=0.68 °°°
**LTASmean (text)**	t(50)=-3.9	p=0.01*	d=0.35°	-73.60	p=0.01*	$_{std}$es=1.08	-	-	-
**LTASstd (text)**	-	-	-	-	-	-	-	-	-
**LTAS kurt (text)**	-	-	-	108.76	p=0.03*	$_{std}$es=1.04	-	-	-
**LTASskew (text)**	-	-	-	2.91	p=0.003**	$_{std}$es=1.179	-	-	-

Abbreviations: PD - Parkinson's disease; HC – Healthy Controls; sd – standard deviation; HNR: Harmonics-to-noise ratio; VOT: Voice to onset time; RST: Rate of speech timing; Int kurt: Kurtosis of Intensity; Int skew: Skewness of intensity; LTAS: Long-term averaged spectrum; sd: Standard deviation; kurt: Kurtosis; skew: skewness; *p<0.05, **p<0.01, ***p<0.001; $^{\Delta}$ - non-significant trend; ° – small effect size, °° – medium effect size, °°° – large effect size; d - Cohen's d effect size, r - Wilcoxon effect size, $_{std}$es – standardized effect size. † - results of multivariable linear regression models adjusting speech variables for age, gender and global cognition (MoCA).

Supplementary Figures

Supplementary Figure 1. Detailed flowchart of patient's recruitment.

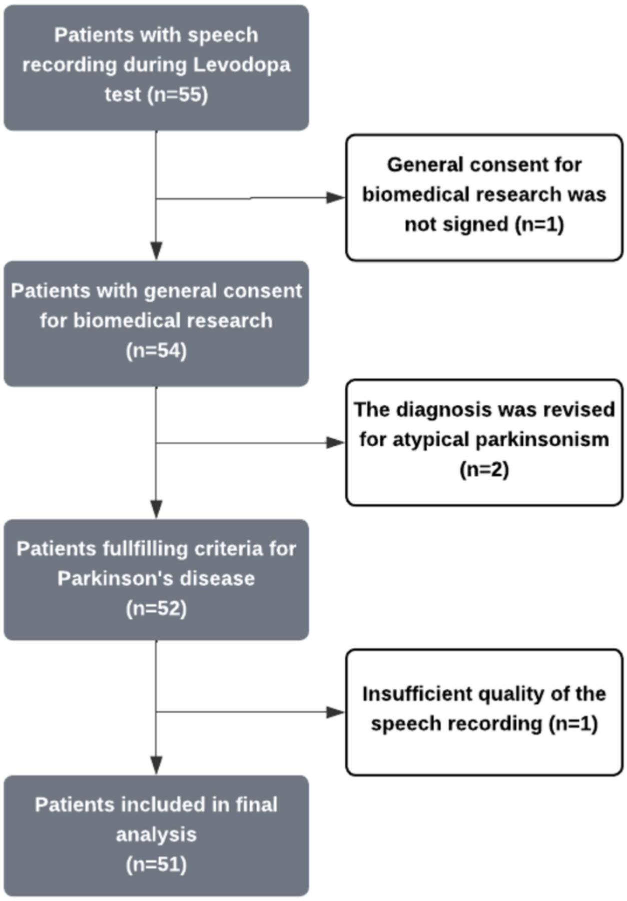

Supplementary Figure 2. Consistency of Individual Digital Speech Biomarkers Across Repeated OFF Medication Recordings. (A-P) Correlation plots of each individual digital acoustic speech biomarker of 10 PD patients repeating the speech protocol 2 times in OFF medication condition 15 minutes apart.

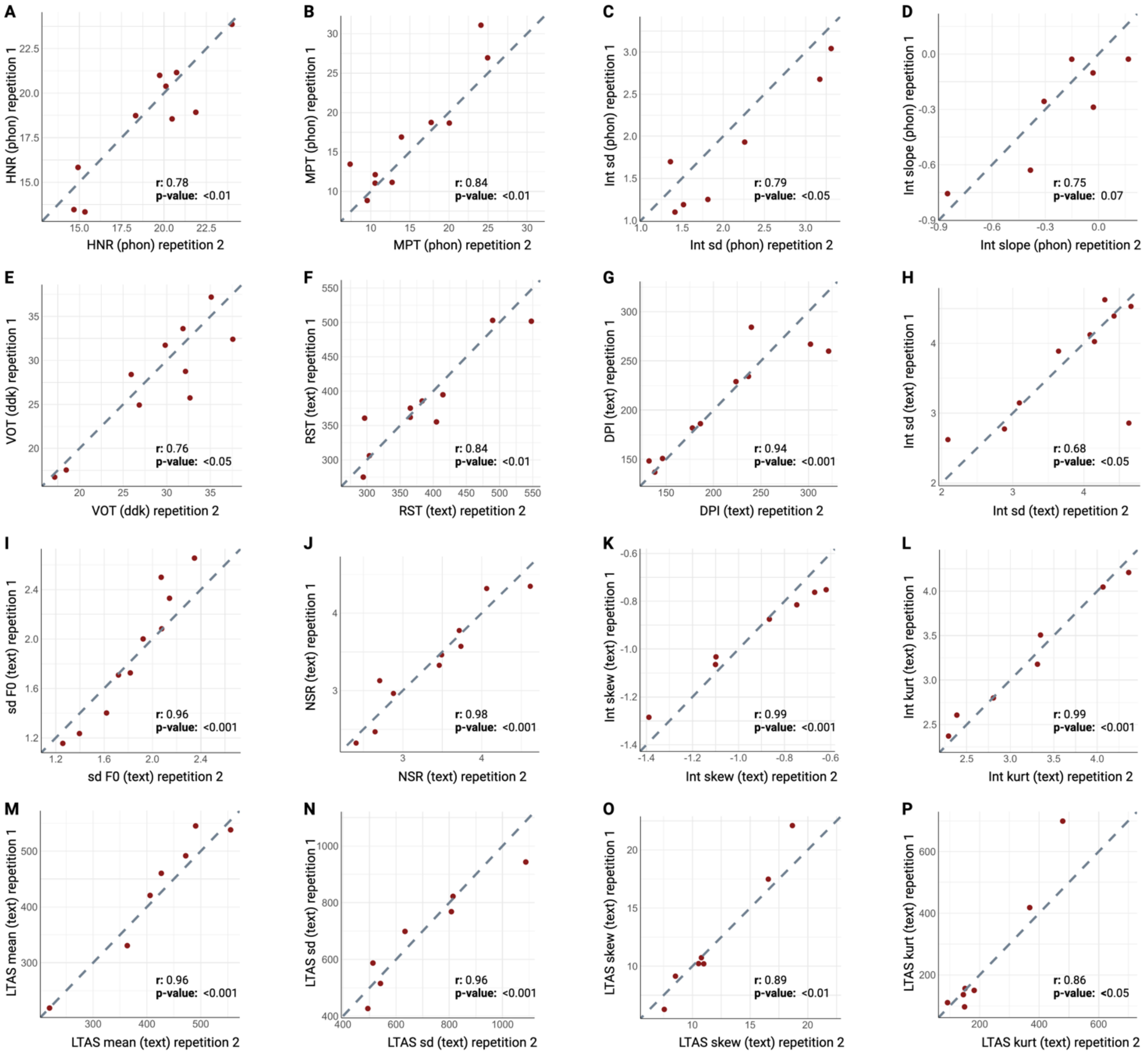

Abbreviations: HNR - Harmonics-to-noise ratio; VOT - Voice to onset time; NSR - Net speech rate; RST - Rate of speech timing; DPI - Duration of pause intervals; Int sd - Standard deviation of intensity; Int Kurt - Kurtosis of intensity; Int skew - Skewness of intensity; sdF0 - Standard deviation of fundamental frequency; MPT - Maximal phonation time; Int slope - Slope of intensity; LTAS - Long-term averaged spectrum; sd - Standard deviation; kurt - Kurtosis; skew - Skewness.

Supplementary Figure 3. Accuracy to predict the total MDS-UPDRS-III score using the selected hypokinetic digital speech biomarkers, the hypokinetic compound speech score and correlation of these predictions with clinical score. (A) Actual value of the change in total MDS-UPDRS-III (grey) vs the predicted value using the selected hypokinetic digital speech biomarkers and respective 95% CI (blue). (A) Actual value of the change in total MDS-UPDRS-III (grey) vs the predicted value using only the hypokinetic speech compound score and respective 95% CI (blue). (C) Correlation plot between change in the hypokinetic compound score and change in the total MDS-UPDRS-III.

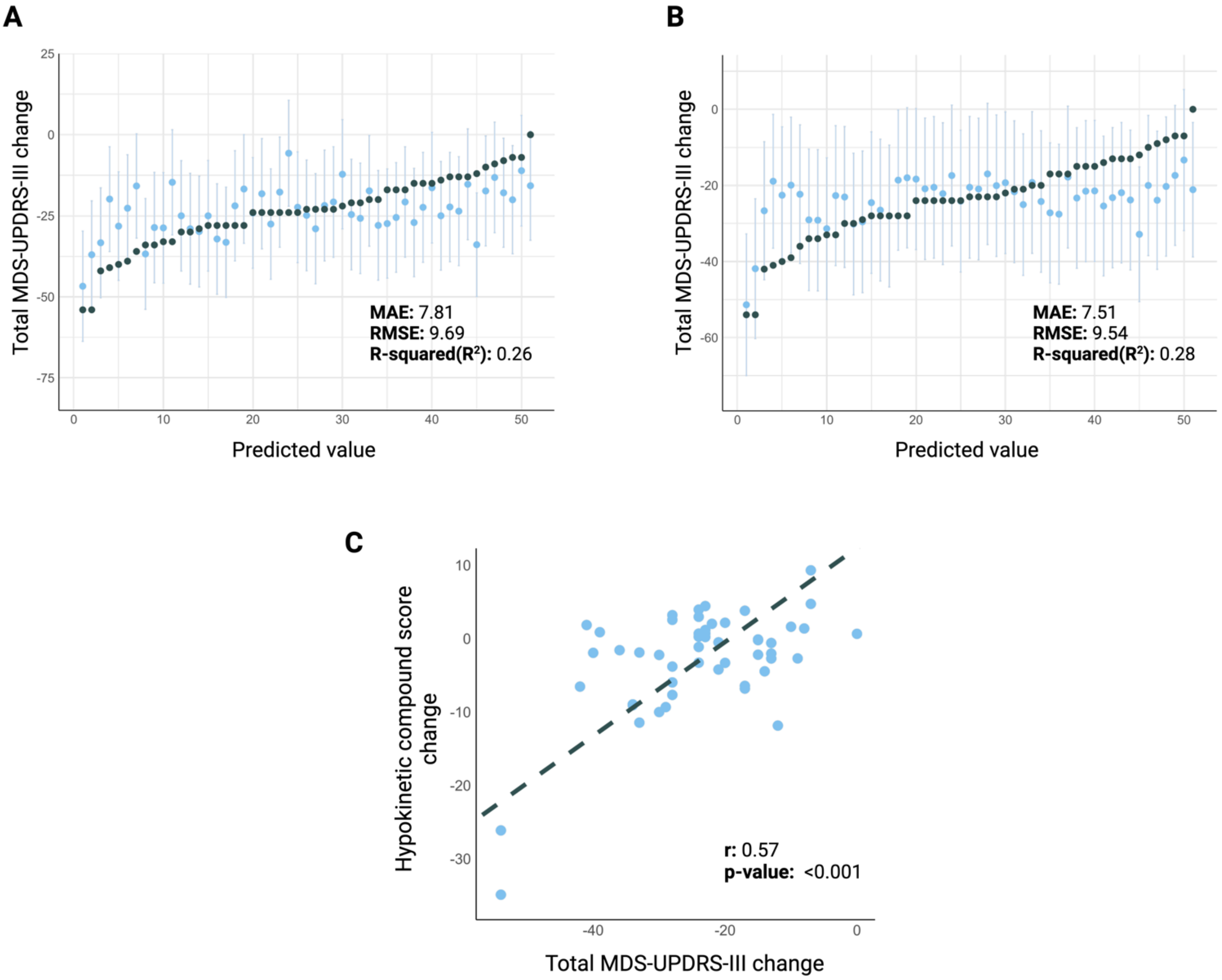

Abbreviations: CI - Confidence Interval; MAE - Mean absolute error; RMSE - Root mean squared error.

Supplementary Figure 4. Correlation Analysis of Clinical Variables and individual digital speech biomarkers. Heatmap depicting the correlations between the change of each individual digital acoustic speech biomarkers and change of the motor outcomes (hypokinetic and hyperkinetic) used in the study. Within each square the Spearman correlation value (r) is presented. Correlations with a p-value <0.05 are depicted in bold.

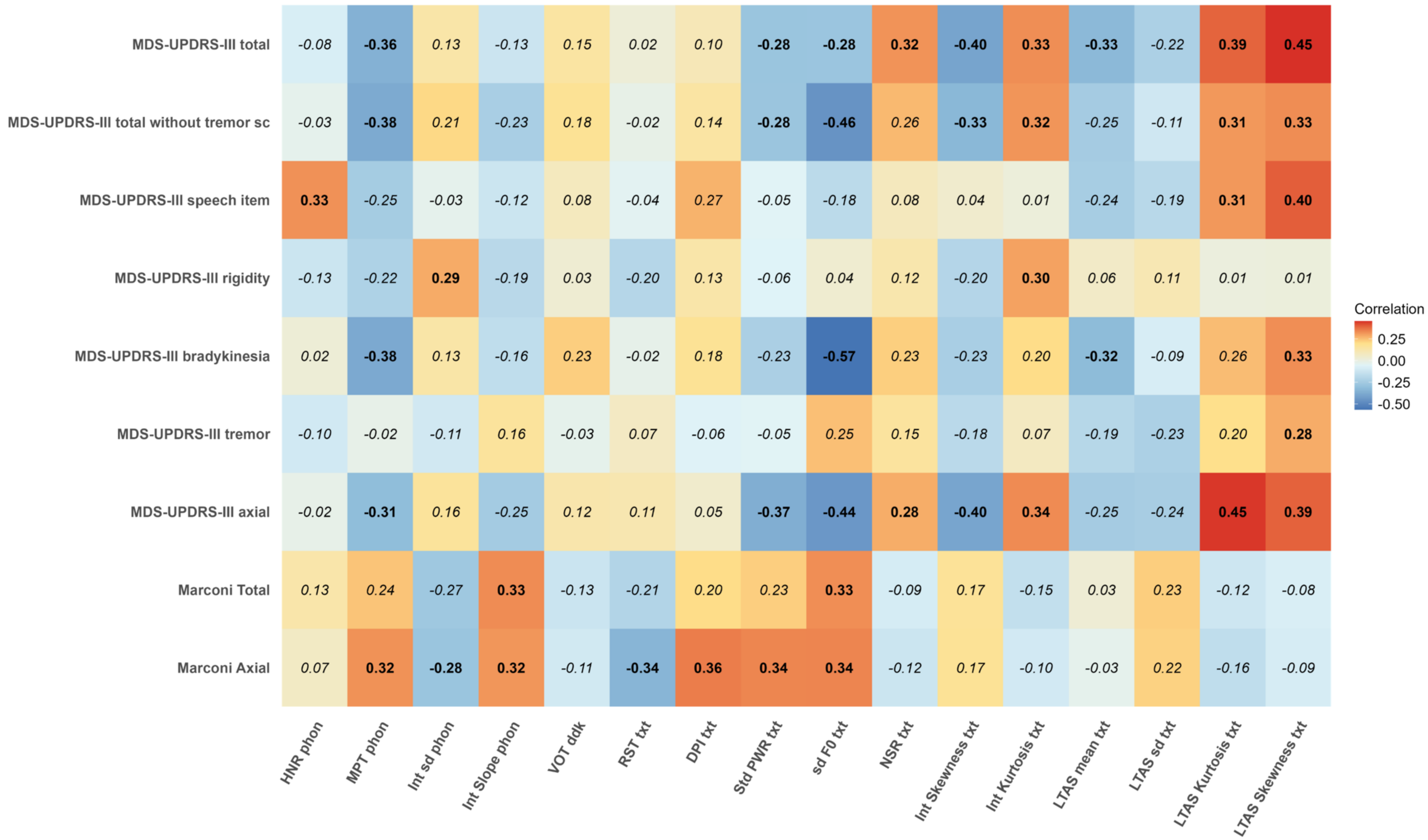

Abbreviations: HNR - Harmonics-to-noise ratio; VOT - Voice to onset time; NSR - Net speech rate; RST - Rate of speech timing; DPI - Duration of pause intervals; Int sd - Standard deviation of intensity; Int kurt - Kurtosis of intensity; Int skew - Skewness of intensity; sdF0 - Standard deviation of fundamental frequency; MPT - Maximal phonation time; Int slope - slope of intensity; LTAS - Long-term averaged spectrum; sd - Standard deviation; kurt - Kurtosis; skew - Skewness. MDS-UPDRS-III - Movement Disorder Society - Unified Parkinson's Disease Rating Scale motor score.

Video Captions

Supplementary Video 1. Application of automated speech analysis in detecting speech abnormalities and predicting overall hypokinetic motor symptom changes.

This representative example demonstrates the speech assessment protocol used in both medication OFF and ON conditions. It includes a segment of the reading task performed by the same PD patient under both conditions. Despite a significant motor response (MDS-UPDRS-III change of 76%), it is possible to appreciate that it can be challenging to perceptually differentiate the medication states. However, our comprehensive speech analysis method identifies clear-cut changes in various speech features (polar graph), such as monopitch (sd F0), monoloudness (Int sd), and skewness of intensity (Int skew). The final segment illustrates the accuracy of our model in predicting hypokinetic symptom changes using the most salient hypokinetic speech features for this individual patient.

Abbreviations: HNR (Harmonics-to-noise ratio), VOT (Voice to onset time), NSR (Net speech rate), RST (Rate of speech timing), DPI (Duration of pause intervals), Int sd (Standard deviation of intensity), Int kurt (Kurtosis of intensity), Int skew (Skewness of intensity), sd F0 (Standard deviation of fundamental frequency), MPT (Maximal phonation time), Int slope (Slope of intensity), MDS-UPDRS-III (Movement Disorder Society - Unified Parkinson's Disease Rating Scale motor score).
